# Hematopoietic stem cell a reservoir of innate immune memory

**DOI:** 10.3389/fimmu.2024.1491729

**Published:** 2024-12-10

**Authors:** Lucas Ruffinatto, Yann Groult, Johanna Iacono, Sandrine Sarrazin, Bérengère de Laval

**Affiliations:** Aix Marseille University, Centre National de la Recherche Scientifique (CNRS), Institut National de la Santé et de la Recherche Médicale (INSERM), Centre d’Immunologie de Marseille-Luminy (CIML), Marseille, France

**Keywords:** hematopoietic stem and progenitor cells (HSPCs), innate immune memory, inflammation, epigenetic, emergency hematopoiesis, metabolism, myelopoiesis

## Abstract

Hematopoietic stem cells (HSCs) are a rare, long-lived and multipotent population that give rise to majority of blood cells and some tissue-resident immune cells. There is growing evidence that inflammatory stimuli can trigger persistent reprogramming in HSCs that enhances or inhibits the cellular functions of these HSCs and their progeny in response to subsequent infections. This newly discovered property makes HSCs a reservoir for innate immune memory. The molecular mechanisms underlying innate immune memory in HSCs are similar to those observed in innate immune cells, although their full elucidation is still pending. In this review, we examine the current state of knowledge on how an inflammatory response leads to reprogramming of HSCs. Understanding the full spectrum of consequences of reshaping early hematopoiesis is critical for assessing the potential benefits and risks under physiological and pathological conditions.

## 1 Introduction

**Figure 1:**
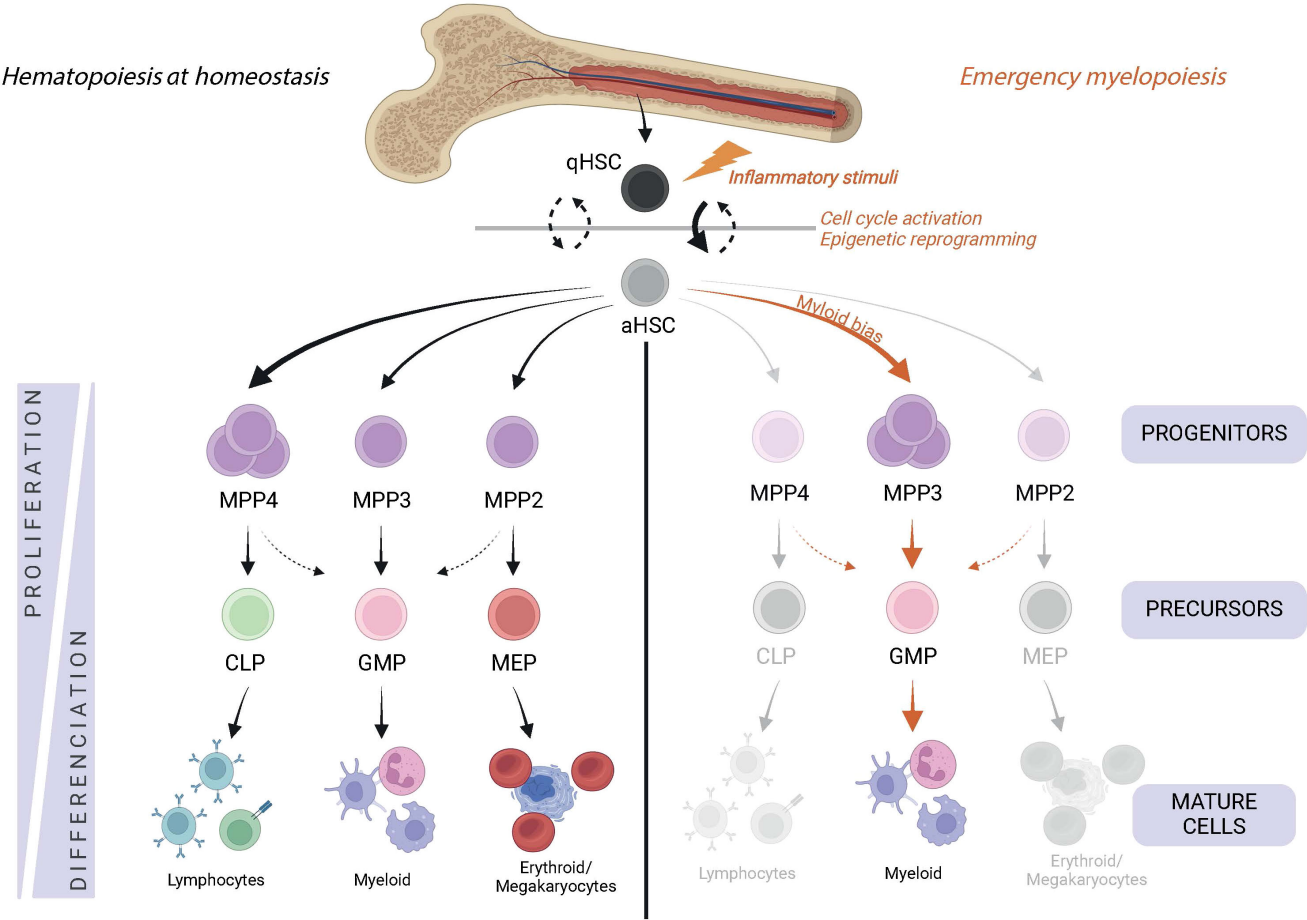
Hematopoiesis during homeostasis and in response to an inflammatory environment. Hematopoietic stem cells (Lin-cKit+ sca-1+ FIt3- CD150+CD48-) are at the top of hematopoiesis and switch from the quiescent state (qHSC) to the activated state (aHSC) to meet the demand for mature cells. They differentiate into multipotent cells (MPP), which have a preference for differentiating into a specific cell lineage. Thus, MPP2 (Lin- cKit+ sca-1+ FIt3- CD150+ CD48+) are oriented towards the erythro-megakaryocytic lineage, MPP3 (Lin- cKit+ sca-1+ Fit3- CD150- CD48+) towards the myeloid lineage and MPP4 (Lin- cKit+ sca-1+ Flt3+) towards the lymphoid lineage. MPP differentiate into the precursors of the MEP (Lin- cKit+ sca-1- CD16/32-CD34-), GMP (Lin- cKit+ sca-1- CD16/32+ CD34+) and CLP (Lin- cKit+ sca-1- FIt3+ IL7R+) lineages, which eventually give rise to mature cells. In response to inflammatory stimuli, HSCs are strongly activated and the myeloid differentiation pathway is mainly favored.

Hematopoietic stem cells (HSCs) are rare, long-lived and multipotent cells. In homeostasis, HSCs oscillate between a quiescent state which allows their maintenance, and an activated state, which allows them to self-renew and produce mature hematopoietic cells (1). Although it is still controversial whether LT-HSCs (Long term HSCs) actively contribute to hematopoiesis at steady state (2–4), the differentiation of HSCs is a continuous process (5) that is finely controlled to avoid a lack of cells (aplasia) or their overproduction (**Figure 1**). As they mature, they gradually lose their long-term activity and multipotency to increase their differentiation capacity. HSCs initially differentiate into ST-HSCs (Short Term HSCs) and MPPs (Multipotent Progenitors), all of which have a limited self-renewal capacity. MPPs can differentiate into all types of mature progenitor cells, each of which has a specific lineage potential. Thus, MPP2, MMP3 and MPP4 are respectively mixed meg-erythroid and myeloid, myeloid and lymphoid biased (6, 7).

**Figure 2:**
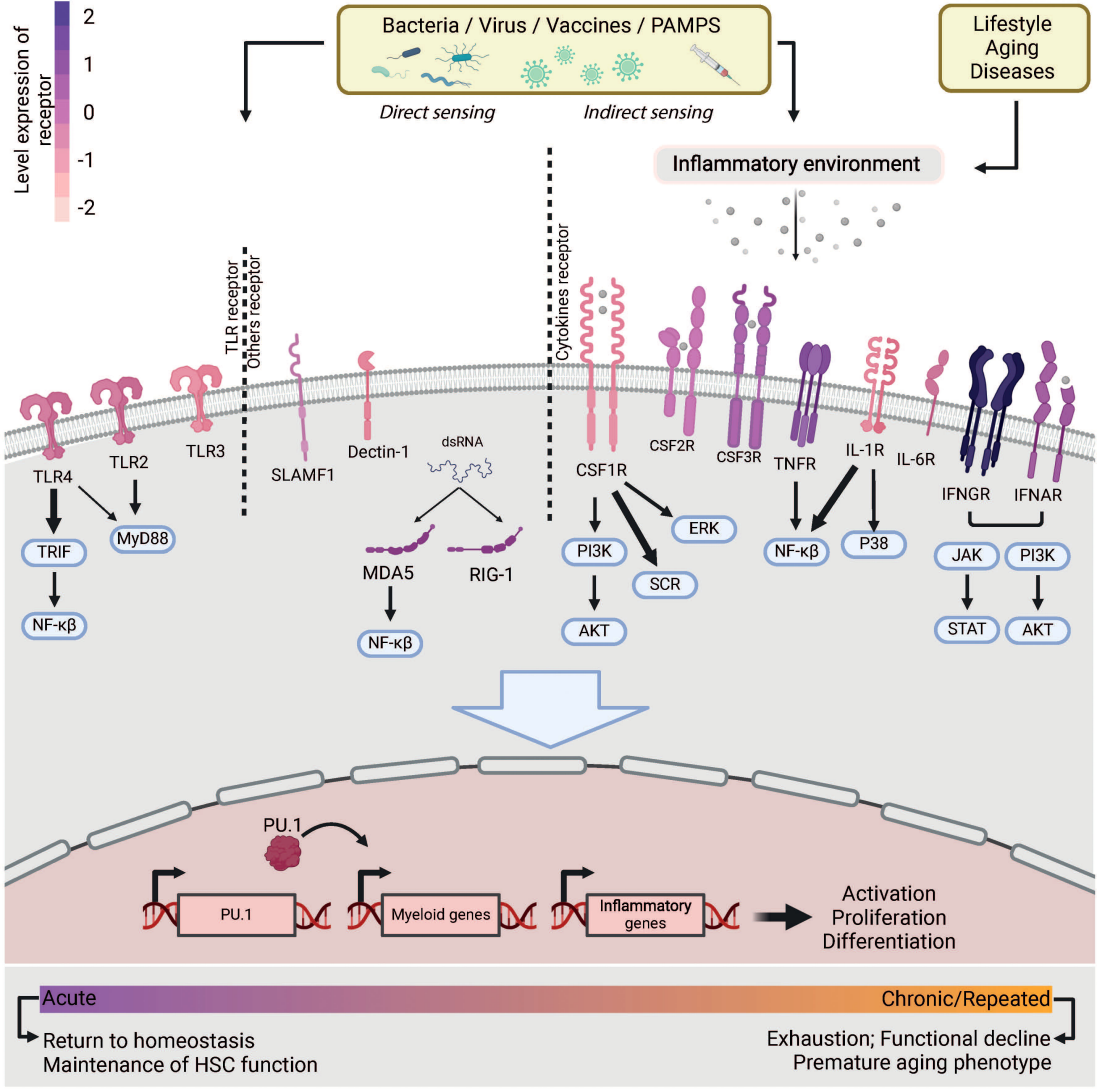
Infection sensing of HSCs. Microbes or an inflammatory environment caused by lifestyle, disease or aging can be recognized by HSCs via receptors they express. The receptors are colored according to their expression levels observed by RNA-seq in HSCs. This sensing triggers a cascade of signaling pathways that lead to the expression of myeloid genes involved in myeloid differentiation or inflammation. The activation of HSCs is maintained for the duration of the stimulation. With chronic or repeated stimulation, HSCs show loss of function and exhaustion.

Various external aggressions can cause a disruption of hematopoietic homeostasis. These include infections or inflammation that cause a high demand for innate immune cells at the site of injury or infection, leading to rapid depletion of these mature effector cells. The body’s response to meet this demand is called emergency hematopoiesis and is initiated at the top of the hematopoietic hierarchy. HSCs themselves initiate this response by directly sensing specific pathogens or inflammatory cytokines, leading to their proliferation and differentiation (**Figure 2**) (8–11). In recent years, some of these signals have been shown to induce innate immune memory (IIM) in HSCs defined by persistent epigenetic reprogramming allowing an adapted response to a secondary insult (12–14). This newly described immunological property of HSCs is called “central trained immunity” and can be transferred to mature cells such as monocytes and macrophages during differentiation (12).

In this review, we describe the infectious and inflammatory signals to which HSCs can respond and how these signals trigger IIM. We will address the mechanisms that lead to the establishment of this memory and its beneficial and detrimental consequences. Thus, we will present the evidence for the positioning of HSCs within the immune system, particularly as a reservoir for IIM.

## 2 HSC sensing of inflammation and infection

Although the role of HSCs in maintaining hematopoiesis in homeostasis is still debated, their role in emergency hematopoiesis is well established. Recent research shows that HSCs are not, as previously thought, insensitive to infection and inflammation. Instead, they are very active in initiating emergency myelopoiesis to produce more immune cells. HSCs express a variety of receptors, including TLRs (Toll Like Receptors) and receptors for inflammatory cytokines, which enable them to recognize infections directly or indirectly via PAMPs (Pathogen-Associated Molecular Patterns) or cytokines (**Figure 2**). Even at homeostasis, HSCs are constantly stimulated by low doses of inflammation, as evidenced by the reduced myeloid production in mice lacking some of these receptors (15). Moreover, recognition of inflammatory cytokines is essential for the emergence and proliferation of HSCs during embryogenesis (reviewed in (16)). Overall, this underscores the importance of inflammatory stimuli recognition by HSCs for the maintenance of homeostasis. This first section provides a comprehensive overview of the stimuli that are directly or indirectly recognized by HSCs.

### 2.1 Inflammatory cytokines

Cytokines are produced by various cells, including immune and non-immune cells, not only in response to infections, as described in the next sections, but also in response to an inflammatory environment caused by a lifestyle such as the Western diet (17) or inflammatory diseases (18), cancers (19), or aging. Recent studies emphasize the susceptibility of HSCs to these molecules. This section focuses on inflammatory cytokines that have been shown to directly affect HSCs.

Type I (IFN-α and β) and type II (IFN-γ) interferons are a subclass of cytokines involved in the antiviral response and were among the first inflammatory cytokines to be studied for their ability to activate HSCs. Indeed, *in vivo* injections of IFN-α and IFN-γ in mice lead to transient JAK/STAT pathway-driven HSC proliferation and their transcriptomic reprogramming, characterized by the expression of interferon response genes (ISG) and myeloid and megakaryocyte-specific genes (9, 15, 20–22). In mice, chronic IFN-α stimulation by daily injections impairs the ability of HSCs to regenerate blood cells (9, 23). This is despite the fact that they return to quiescence by avoiding p53-dependent apoptosis and limiting the depletion of HSCs normally observed in chronic infections (23). Analysis of IFN-α-deficient HSCs in chimera mice has shown that much part of the effect of IFN-α is due to its direct recognition by HSCs (9). This is supported by the high IFNAR expression on the surface of HSCs (**Figure 2**).

Cytokines are a broad-spectrum of signaling molecules that regulate the immune response by enabling cell communication. The proinflammatory cytokines IL-1β, IL-6 and TNF-α, which trigger inflammation, have been shown to activate HSCs and induce their myeloid differentiation. For instance, IL-1β and TNF-α drive the transient proliferation of HSCs by increasing the expression of the myeloid master transcription factor PU.1, which is largely mediated by the NF-κB pathway (10, 24). While TNF-α drives apoptosis in GMP, HSCs have developed a NF-κB-dependent survival mechanism against TNF-α. Under inflammatory stress induced by various microbial challenges or 5-FU-induced myeloablation, TNF-α expression in the niche is essential for HSC survival and subsequent myeloid regeneration (24). IL-6 is less studied and it is not known how IL-6 injections affect HSCs, but blocking IL-6 signaling inhibits expression of inflammatory genes in HSCs in response to LPS during neutropenia (25) or in response to murine hepatitis virus 1 (MHV-1) in mice or SARS-CoV2 in humans (26).

Other cytokines known as growth factors activate the HSCs. M-CSF transiently promotes the conversion of murine HSCs into myeloid cells through direct activation of the myeloid master transcription factor PU.1, which mainly depends on Src, ERK and PI3K signaling downstream of the M-CSF receptor (27). G-CSF treatment also promotes myelopoiesis through the proliferation of myeloid CD41+ HSCs as well as emergency megakaryopoiesis by inducing the emergence of an HSC population biased toward megakaryocytes production (28). In addition, G-CSF triggers the mobilization of dormant CD41- HSCs in the blood, liver and spleen, a process called extramedullary hematopoiesis. Although these HSCs have been shown not to proliferate (28), they can highly contribute to emergency myelopoiesis by giving rise to large numbers of neutrophils and monocytes (29). The direct effect of GM-CSF on HSCs has been poorly studied and the results are conflicting. A study in which sorted cells were analyzed after *in vitro* exposure to GM-CSF has shown that GM-CSF is recognized by ST-HSCs and MPPs, but not by LT-HSCs (30). However, blocking the GM-CSF receptor in response to β-glucan or inflammatory diseases such as spondylarthritis, impairs the expansion and mobilization of HSCs in peripheral organs, suggesting that they may recognize GM-CSF (13, 30).

### 2.2 PAMPS

PAMPS (Pathogen-associated molecular patterns) are molecules produced mainly by bacteria or viruses whose conserved motifs are recognized by specific PRRs (Pathogens Recognition Receptors) expressed on immune cells. Among these receptors, the TLRs (Toll-Like Receptors) were the first to be discovered to be expressed on the surface of HSCs (**Figure 2**), particularly TLR4 and TLR2, indicating for the first time an interaction between microbial particles and HSCs (8). Many studies have investigated the effects of LPS and PAM3CSK4, bacterial PAMPs recognized by TLR4 and TLR2, respectively, on HSCs (8, 14, 25, 31, 32). *In vitro* exposure of mouse HSC to LPS or PAM3CSK4 instruct their myeloid differentiation and production of inflammatory cytokines (8, 25). Chimeric mice containing a mixture of BM from TLR4-deficient and WT BM allow the analysis of TLR4-deficient HSCs in a WT environment. This shows that proliferation of TLR4-deficient HSCs is reduced in response to LPS, demonstrating at least a partial direct sensing of LPS on HSCs (31, 33). Activation of TLR4 initiates two signaling pathways via the adaptor proteins Myd88 and TRIF. Recognition of LPS by HSCs leads to their transient activation and expansion via the TRIF signaling pathway, but not via Myd88, which promotes myeloid (8) and megakaryocytic differentiation (5) while reducing their reconstitution capacity (31, 33). Although there is evidence for direct recognition of LPS and PAM3CSK4 by HSCs, these signals may also be sensed via non-cell-autonomous pathways involving inflammatory cytokines produced by the microenvironment in response to PAMPs. Indeed, mice lacking the receptors for IFN-α, IFN-γ, TNF-α, IL-1β or IL-6 showed reduced LPS-induced HSC proliferation or myeloid differentiation, suggesting that these cytokines play a role in the activation of HSCs following *in vivo* LPS exposure (25, 33). In addition, G-CSF is also produced in response to LPS to induce HSCs mobilization (29). Similarly, HSC activation by PAM3CSK4 is also mainly mediated by both G-CSF and TNF-α, but not by IFN-γ (32).

Although other PAMPs or analogs that mimic viral infection such as polyI:C (dsRNA analogs) and R848 (resiquimod) or compounds derived from yeast and fungal walls (β-glucan) act on HSCs, there is no clear evidence expression – despite weak transcriptomic expression as shown in the **Figure 2** – of their corresponding receptors (TLR3 or TRL7/TLR8 or Dectin-1, respectively) on murine HSCs, suggesting that they are only indirectly recognized. These viral analogs cause transient proliferation, mobilization, myeloid and megakaryocytic differentiation of HSCs (2, 9, 21, 34, 35). In addition, HSCs function is impaired during the acute response or chronic stimulation, as they do not restore a myelodepleted bone marrow (9, 34, 35). In mice lacking the IFN-α receptor or TNF-α, polyI:C does not fully activate HSCs, suggesting that the effect of polyI:C is mainly mediated by IFN-α and TNF-α (9, 24, 35). β-glucan tends to have a chronic effect as it remains in the organism for months. It has been shown to primarily activate myeloid CD41+ HSCs via IL-1β or GM-CSF, leading to long-lasting expansion and sustained reprogramming of HSCs, with a myeloid bias that persists for at least 28 days after injection (13).

Human CD34+ hematopoietic stem/progenitor cells (HSPC) express TLR4, TRL2, TLR7/TRL8 and TRL9. *In vitro* exposure of these cells to their respective agonists (LPS, PAM3CSK4, R848 and CPG DNA) instructs their myeloid differentiation (36–38).

### 2.3 Pathogens

Bacteria, viruses and yeasts can also be recognized by HSCs. Although direct detection via PAMPs or microbial particles is possible, numerous studies have emphasized the importance of indirect detection, through inflammatory cytokines produced by the microenvironment in response to the microorganism.

#### 2.3.1 Indirect detection

Viral lung infections such as influenza and SARS-Cov2 have been shown to affect HSC, both in Mouse and Human (26, 39). Influenza induces activation and proliferation of murine HSCs during the infection period (approximately 14 days), allowing emergency myelopoiesis and megakaryopoiesis. Analysis of mice lacking the major cytokines produced in response to the virus showed that HSC activation is dependent on IL-1β in the early phase and IL-6 in the later phase of infection (39). Analysis of HSCs in the blood of patients at different time points after severe COVID-19 recovery shows a persistent myeloid signature indicative of myeloid differentiation. This is confirmed by the increase in the number of myeloid progenitors that persist one month after disease onset (26). Blocking IL-6 signaling in patients with Tocilizumab, a monoclonal antibody targeting the IL-6 receptor, decreases the expression of inflammatory genes in HSCs, confirming the indirect activation of HSCs by SARS-CoV2 through sustained production of IL6 (26). Similar observations were made in response to murine hepatitis virus 1 (MHV-1), confirming a role for IL-6 in the activation of murine HSCs in response to viral lung disease (26). Another example is LCMV (lymphocytic choriomeningitis virus), which, in contrast to influenza and SARS-CoV2, causes a transient loss of murine HSCs through increased apoptosis and also permanent functional impairment (35, 40). In chimera mice, HSCs lacking the IFN-γ receptor were less activated by infection than WT HSCs (35). Furthermore, blocking IFN-α and IFN-γ signaling during infection prevents loss of HSC function (40). IFN-γ also activates HSCs indirectly by promoting the production of IL-6 by mesenchymal stem cells of the BM niche (41). All these studies suggest indirect recognition of these viral infections by HSCs.

Mycobacterial infections lead to chronic infections. In response to infection with *Mycobacterium avium*, HSCs exit dormancy and begin to proliferate and undergo myeloid differentiation. Further analysis has shown that IFN-γ and Stat proteins are involved in this process (22, 42). In contrast, recognition of *Mycobacterium tuberculosis* by HSCs depends mainly on IFN-α although IFN-γ is also expressed. Albeit systemic and pulmonary infection with *Mycobacterium tuberculosis* triggers an interferon response in HSCs, it does not induce their expansion or myeloid differentiation, but rather myeloid suppression through induction of necroptosis in myeloid progenitors mediated by IFN-α (43). Another example is systemic infection with *Escherichia coli*, which transiently alters HSC functions and induces their mobilization in the spleen dependent on the G-CSF and CXCL12 pathways (29).

#### 2.3.2 Direct detection

All studies described so far have emphasized indirect sensing of bacteria by inflammatory cytokines. However, there is also evidence for direct sensing of live pathogens by HSCs. We have shown that *Brucella abortus*, a gram-negative coccobacillus, can instruct myeloid differentiation of HSCs through a direct interaction between the cell surface protein CD150 (SLAMF1) on HSCs and the outer membrane protein Omp25 on the bacterium (11). While CD150 is currently the best marker for the identification of HSCs, its function in HSCs was previously unknown. We demonstrated that this receptor can sense the bacterium without allowing its entry into HSCs. Using a reporter mouse model of early myeloid commitment, we found that interaction between CD150 and Omp25 triggers functional commitment of HSCs in the bone marrow towards the myeloid lineage, leading to increased production of downstream myeloid progenitors and mature cells. Specifically, mice lacking CD150 and those infected with a *B. abortus* strain lacking Omp25 showed reduced expression of the myeloid master transcription factor PU.1 in HSCs, thereby impairing emergency myelopoiesis.

HSC may also directly sense *Candida albicans*, a fungus that causes opportunistic infections. Injection of this microorganism into mice a few days after transplantation of TLR2-deficient HSPCs failed to induce expansion and differentiation of the transplanted cells into myeloid cells. This finding indicates that direct recognition of *C. albicans* by TLR2 on HSCs is essential for these developmental processes (44). Similar observations were made *in vitro* when HSPCs were stimulated with *C. albicans.* This stimulation induced proliferation and differentiation, but these effects were not observed in the absence of Myd88 signaling (45). Since HSCs express TLR2 and can recognize PAMPs via TLR2, it is plausible that they might also directly recognize *Candida albicans*.

#### 2.3.3 Infection of HSCs

Although some pathogens such as LCMV, *Mycobacterium tuberculosis* and *Brucella abortus* have been found in bone marrow, they are not capable of infecting murine HSCs (11, 40, 43). In Human, while some studies show that viruses, such as Epstein-Barr virus (EBV) and human herpes virus 7 (HHV-7), can infect the heterogenous population of CD34+ HSPCs (46, 47), no study has demonstrated that this is the case for more purified HSCs. In the case of chronic active EBV disease (CAEBV), EBV-infected HSPCs show a reduced stem cell signature and increased myeloid differentiation (47). Similarly, *in vitro* infection of human CD34+ cells with HHV-7 promotes their differentiation along granulocytic and erythroid lineages. In addition, viruses were detected in mature myeloid cells (46), indicating that direct infection of HSPCs may contribute to disease persistence.

### 2.4 Vaccines

The BCG vaccine against tuberculosis is an attenuated strain of *Mycobacterium bovis*. When BCG reaches the bone marrow, it triggers a myeloid program in the HSCs and causes a long-lasting activation and expansion that can be observed in mice for up to 150 days after injection. In mice, this proliferation is dependent on IFN-γ, as HSPCs lacking this receptor hardly respond to BCG (12, 43). Transcriptomic analysis of human CD34+ HSCs after BCG vaccination shows an enrichment for myeloid-specific genes 90 days after vaccination, suggesting their commitment to myeloid lineages and confirmed by the observation of increased number of granulocytes in the blood of patients (48).

As mentioned above, sensing of inflammatory stimuli by HSCs leads to their activation, characterized by transcriptomic rewiring inducing an exit from quiescence, their proliferation and differentiation towards the myeloid and erythroid lineages. Inflammation drives the rapid myeloid differentiation of HSCs by increasing the proliferation of each progenitor subtype (49). Emergency megakaryopoiesis occurs differently by bypassing the differentiation steps. Indeed, megakaryocytes can arise directly from HSCs in response to inflammation (21, 28, 39). Further investigations are needed to determine whether this is a global response to inflammation.

Interestingly, HSPCs themselves secrete TNF-α and IL-6 upon *ex vivo* stimulation with LPS and PAM3CSK4 (25). Recently, this finding has been refined by the discovery of a specific subpopulation of myeloid MPPs with a secretory phenotype. This subset is increased in response to LPS and PAM3CSK4 and promotes rapid myeloid differentiation of HSCs through a local production of IL-6 and TNF-α (50).

In addition to their immediate response to stimulation, HSCs are able to store it in a process called innate immune memory (IIM), which we describe in the following section.

## 3 Innate immune memory

Immunological memory was defined as a process by which information from a first infection is stored, enabling a faster and more effective response to a second infection. Initially, its study was limited to the adaptive component of immunity, a defense mechanism specific to jawed vertebrates. Adaptive immune memory is supported by the specific recognition of antigens by TCR and BCR receptors. The diversity of these receptors is the result of random genetic recombination, which allows the formation of an almost unlimited reservoir of receptors. More recently, it has been shown that part of memory also depends on the innate component of immunity, a highly conserved mechanism of host defense shared by plants, invertebrates and vertebrates throughout evolution.

In contrast to adaptive immune memory, which is achieved through genetic recombination, innate immune memory (IIM) is established through metabolic and epigenetic reprogramming. This reprogramming results in enhanced or dampened innate immune functions of cells, which can be beneficial or detrimental depending on the context.

**Figure 3:**
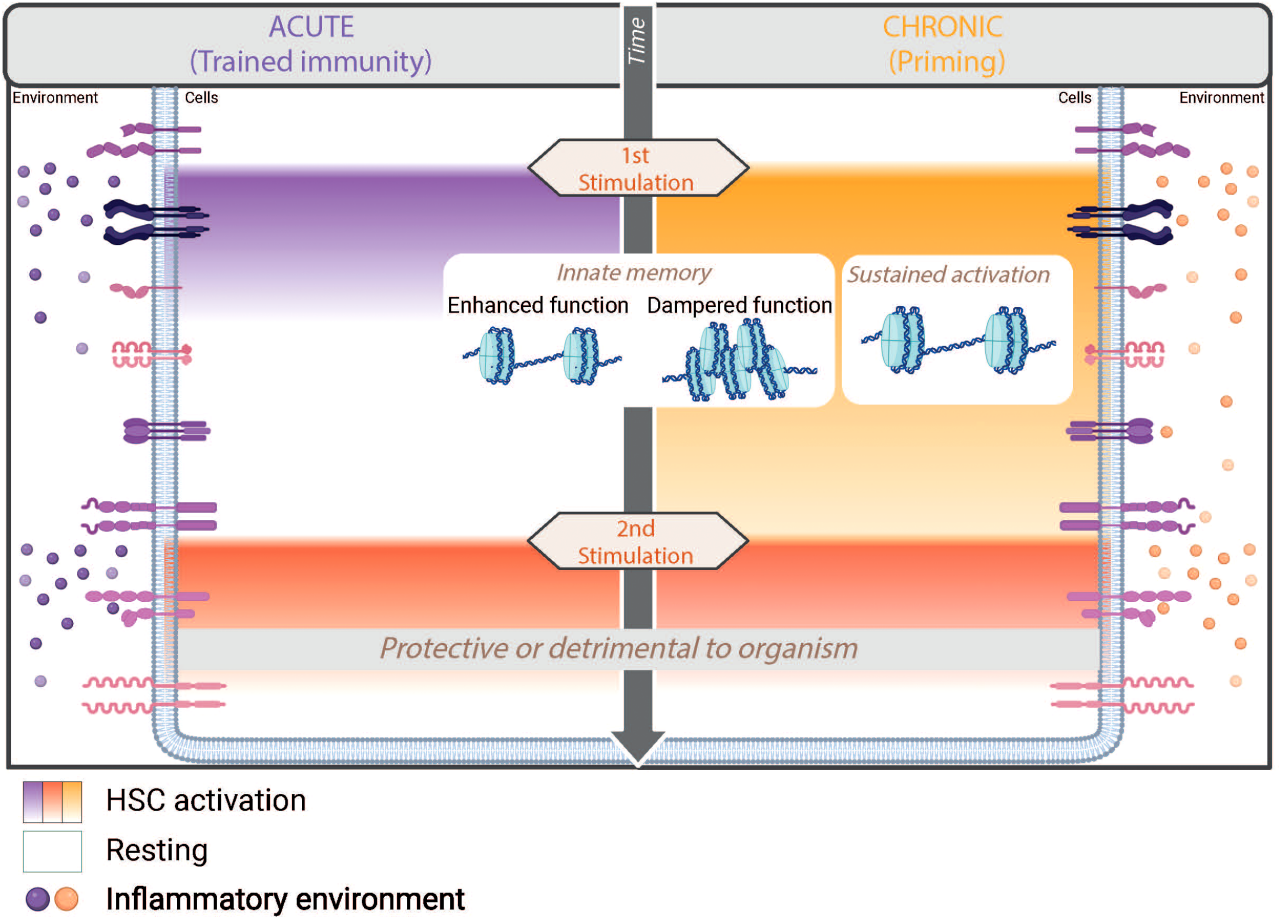
Innate immune memory programs triggered by acute and chronic inflammation. IIM is a defense mechanism of the body that provides greater protection against secondary insults thanks to epigenetic reprogramming that enables enhanced and/or dampened functionality of cells. In hematopoietic stem cells (HSCs), various IIM programs - trained immunity, priming or tolerance - can be induced depending on the context of the infection. In the bone marrow, the transient pro-inflammatory environment induced by acute stimulation leads to transient activation of HSCs but long-term trained immunity. Conversely, chronic stimulation maintains a pro-inflammatory environment that leads to sustained activation of HSCs and priming. In both cases, IIM leads to enhanced function upon second stimulation, while tolerance shows a dampened response. Although IIM provides clear protection against many secondary insults, it can also have unfavorable consequences for the organism.

Nowadays, the IIM is divided into three main programs: “Trained Immunity”, “Priming” and “Tolerance”. These programs are distinguished by the state of the cells between two stimuli – either returning to a baseline state or maintaining continuous activation – and also by whether they elicit enhanced or suppressed cell function during a secondary response. The memory generated by these programs enables modulation of the cellular response to different challenges and gives the cells a high degree of adaptability (**Figure 3**).

“Trained immunity” describes a program in which the first stimulation triggers a transient activation of the cells, allowing them to return to the basal state before subsequent stimulation. In contrast, “priming” refers to a different IIM program in which the cells do not return to the resting state at the time of the second insult, but instead exhibit a sustained activation phenotype. This persistent activation can be explained either by a too short interval between two stimulations or by the persistence of the stimulus leading to chronic stimulation. In the context of reinfection, both “trained immunity” and “priming” have been shown to allow the establishment of a memory that enables an enhanced response with modulated gene expression and function of immune system. This is in contrast to the third program described in the literature, known as tolerance, which is characterized by a repression of an immune response (51).

Although “trained immunity” and “priming” share similar hallmarks – such as epigenetic modifications that predominantly affect genes involved in immune response and differentiation, increased metabolic capacity, and enhanced functions – the protection observed in subsequent infections could be achieved by different mechanisms for each process. On the one hand, the transient activation observed in “trained immunity” enables epigenetic imprinting that persists over a long period of time and enables reactivated cells to develop an enhanced immune response. On the other hand, the persistent activation of cells during “priming” currently prevents us from clearly distinguishing the role of epigenetic rewiring from the role of the activation state in the protection conferred in response to subsequent infection. To distinguish the effects of memory from the effects of cell activation state on modulating the response to secondary infection, some studies have used an experimental model of bone marrow transplantation in naïve recipients (12, 52). This approach ensured that no stimulated environment was present at the time of the second stimulation, allowing researchers to determine whether the memory carried by the transplanted cells contributed to immunological “priming”.

To understand the role and underlying molecular mechanisms of each of these programs in the induction of IIM, it is also important to consider the type of cells being studied and to keep in mind that cells with the capacity for self-renewal – such as stem cells – are able to switch from activated to non-activated states and likely do not have the same rewiring capacities as mature cells. Finally, it is important to consider that the duration: acute or chronic stimulation and the magnitude of stimulation could modulate the innate immune response.

### 3.1 Central innate immune memory

IIM was first discovered in mature innate cells such as monocytes/macrophages but also in neutrophils, natural killer (NK) cells and innate lymphoid cells (ILCs) as reviewed in (53). Surprisingly, it has been shown that the effects of IIM can last from a few months to years, which is not consistent with the short lifespan of innate immune cells. This finding has led to a shift in focus to the study of long-lived cells such as HSCs and bone marrow progenitor cells (HSPCs). Perception of microbial components and inflammatory molecules by HSCs and HSPCs has been shown to lead to metabolic rewiring and remodeling of their epigenetic landscape (12–14, 52). These studies have also indirectly shown a possible transfer of memory from HSCs and HSPCs to mature innate cells, suggesting that they serve as a reservoir for IIMs. Therefore, the memory carried by HSCs is referred to as central IIM, whereas the IIM induced in mature myeloid cells is referred to as peripheral IIM.

#### 3.1.1 Central IIM programs induced by chronic HSC stimulation

##### 3.1.1.1 IIM induced by Bacillus Calmette-Guerin (BCG) and *Mycobacterium tuberculosis* in HSCs

Epidemiological studies have shown that BCG vaccination significantly boosts innate immunity and protects against a broad spectrum of diseases (54). IIM was first described in monocytes from BCG-vaccinated patients, which showed increased expression of proinflammatory cytokines such as TNF-α, IL-6 and IFN-γ as well as increased clearance capacity in response to secondary infection with *Mycobacterium tuberculosis* or *Candida albicans* (48, 55). Furthermore, these results indicate a cross-protective effect of IIM induced by BCG vaccination.

Kaufman et al. were the first to demonstrate that intravenous injection of BCG in mice induces IFN-γ-dependent IIM in HSCs (12). The persistence of BCG in the bone marrow (at least up to 7 months post-injection) chronically alters the bone marrow microenvironment and leads to a sustained activation and myeloid signature in HSCs, indicating a “priming” program of IIM in both humans and mice (12, 48). Although the direct effect of secondary *Mycobacterium tuberculosis* infection on HSCs has not been analyzed, mice receiving bone marrow from BCG-vaccinated mice showed improved protection against the bacteria months after transplantation. This is also observed in bone marrow-derived macrophages (BMDM) from BCG-vaccinated mice, but not when the mice lack IFN-γ signaling, demonstrating an important role of IFN-γ in the induction of IIM after BCG vaccination (12, 43). Analysis of the epigenetic landscape revealed that BCG vaccination increased H3K27 acetylation marks at innate immune genes and myeloid enhancers in BMDM, confirming that BCG induced epigenetic rewiring and suggesting that this might be acquired from the central IIM (12). Therefore, it would be interesting to also study the epigenome of HSCs to determine whether similar epigenetic modifications were acquired and to evaluate their possible transfer to BMDMs.

*Mycobacterium tuberculosis* also induces a priming program in bone marrow cells, including HSCs. However, in contrast to mice infected with the attenuated BCG strain, macrophages derived from the bone marrow of mice infected with *Mycobacterium tuberculosis* show impaired bacterial clearance upon subsequent infection. This detrimental reprogramming which persist for at least one year, is dependent on chronic IFN-α production in response to chronic infection with *Mycobacterium tuberculosis* (43).

##### 3.1.1.2 β-glucan induced priming in HSCs

β-glucan, a PAMP from *Candida albicans*, has been shown to modulate the immune response in mouse and human monocytes. Indeed, *in vivo* exposure to a low dose of *Candida albicans* or β-glucan protects mice from subsequent lethal *Candida albicans* or *Mycobacterium tuberculosis* infections, demonstrating the presence of IIM. Furthermore, injection of β-glucan resulted in epigenetic and metabolic reprogramming in mouse monocytes with an increase in pro-inflammatory cytokine production and anti-mycobacterial capacity upon subsequent infection (56, 57).

In the early hematopoietic compartment, injection of β-glucan in mice resulted in sustained activation and myeloid skewing of SLAM HSCs (CD48- CD150+) that was still observable at 28 days post-injection, indicating chronic activation of HSCs. This suggests that a “priming” program is ongoing (13). Indeed, metabolic rewiring, an hallmark of IIM, with increased glycolysis was observed in the progenitor cells. Moreover, *in vivo* exposure to β-glucan protects HSCs from DNA damage induced by a secondary stress such as LPS or chemotherapeutic agents and increased the survival rate of mice after repeated 5-FU injections (13). Finally, BMDM of β-glucan stimulated mice showed improved *Mycobacterium tuberculosis* clearance (43). β-glucan also protects against melanoma or lung carcinoma induced by orthotopic injections of tumor cells and effectively limits tumor growth. This is due to epigenetic rewiring of GMP, which enables the production of neutrophils with ROS-dependent antitumor activity. Mice transplanted with bone marrow from β-glucan-stimulated donors show reduced tumor progression for at least 6 weeks post-transplantation. This finding suggests that IIM may occur at the level of early hematopoiesis, potentially involving transplanted HSPCs and progenitors (58).

The absence of IL-1β signaling during β-glucan stimulation resulted in a reduction of IIM hallmarks such as glycolysis in progenitor cells and protection against subsequent *Mycobacterium tuberculosis* (13, 57). Another study has shown that IFN-α, which is also produced in response to β-glucan, may be an important trigger of IIM that protects against tumors, as neutrophils lacking the IFN-α receptor failed to reduce tumor growth (58). These findings highlight the important role of the microenvironment in chronic stimulation-induced IIM programs such as HSC priming.

##### 3.1.1.3 *M.avium*-dependent HSCs priming confer central IIM

To analyze IIM specifically in the context of chronic infection by *Mycobacterium avium*, Kain et al. used a model of progenitor transplantation in combination with antibiotic treatment to eliminate the initial infection signal prior transplantation, ensuring analysis of IIM triggered by “priming”. In response to secondary *M. avium* infection, mice that received primed HSPCs show less systemic inflammation but better disease control associated with improved bacterial clearance. BMDM from *M. avium*-stimulated mice showed increased metabolic activity and enhanced phagocytosis upon secondary *M. avium* infection, recapitulating all IIM features (52). Interestingly, memory following *M. avium* infection leads to a decrease in proinflammatory cytokines and peripheral immune cells upon secondary infection with the bacteria, a response often associated with “tolerance” in the literature. In this case, however, it is associated with improved bacterial control and reduced pathogenesis. This suggests that the negative regulation of pro-inflammatory pathways induced by the IIM program may be beneficial in certain situations, such as secondary *M. avium* infection. Remarkably, mice receiving HSPCs pre-stimulated with *M. avium* are also protected from subsequent influenza A virus (IAV) infection. However, in contrast to the responses observed with subsequent *M. avium* infection, these mice exhibited decreased viral load in the lung in conjunction with increased cytokine responses including IFN-γ (52). Thus, the central IIM of HSPCs induced by *M. avium* confers a protective effect, albeit with distinct innate immune features depending on the second infection. Thus, the central IIM of HSPCs induced by *M. avium* confers a protective effect, but with different innate immune system characteristics depending on the second infection. Taken together, these results suggest that a specific central IIM elicited by a particular stimulus leads to different cytokine responses upon re-exposure to different pathogens, consistently resulting in reduced pathogen burden and enhanced protection. It would therefore be important to investigate whether this phenomenon is related to a modulation of the originally acquired memory or whether, depending on the type of pathogen, the activated pathways of the immune response in the cells are different and thus influence the interaction with the established immune memory.

##### 3.1.1.4 Western and high-salt diets

Recently, “inflammatory” diets, such as the Western diet (WD) and the high-salt diet (HSD), have been shown to induce transient sterile systemic inflammation in mice (59), leading to long-lasting changes in the innate immune response, suggesting the induction of central IIM (17, 60).

Interestingly, Christ and colleagues compare the effect of a WD with the effect of a passed WD, allowing the comparison of memory induced by either “priming” or “trained immunity”. *In vitro*, myeloid bone marrow cells sorted from WD-fed mice show higher secretion of inflammatory cytokines in response to PAMPs, even when the mice returned to a normal diet (17). These results indicate that myeloid cells in the bone marrow retain a memory phenotype even after systemic inflammation subsides, suggesting that “trained immunity” may arise in WD-stimulated HSCs.

In mice, both WD and HSD induce myeloid rewiring in bone marrow progenitor cells. This was reflected in an increase in the number of myeloid progenitor cells, GMPs, which correlated with transcriptional changes, including upregulation of genes involved in cell proliferation and myeloid skewing (17). Interestingly, WD-induced transcriptional rewiring persists – although weaker – when returning to normal diet. This can be explained either by a microenvironment in the bone marrow that continuously activates GMPs or by the maintenance or production of qualitatively different GMPs. Hallmarks of IIM such as upregulation of glycolysis and cholesterol biosynthesis pathways and persistent chromatin modifications were also detected, suggesting that WD and HSD enable metabolic and epigenetic rewiring (17, 60). Following *in vivo* LPS exposure, transcriptional changes in GMPs induced by WD synergize with those induced during stimulation and enhance the myeloid response. Interestingly, the LPS response of GMPs from mice that have returned to a normal diet is different, with a lower inflammatory response, suggesting two different types of IIM (17).

Whether the establishment of IIM by these diets is beneficial or detrimental to the organism is still controversial. Indeed, monocyte-derived macrophages from HSD-fed mice failed to resorb brain hematoma after intracerebral hemorrhage, a severe form of stroke, resulting in neurological damage (60). Furthermore, this defect is recapitulated in mice receiving bone marrow cells from HSD-fed mice. Thus, both WD and HSD diets induce IIM that is deleterious in the context of HSD in response to intracerebral hemorrhage. This role of diet-induced IIM needs to be better understood as the consumption of these types of diets has reached epidemic proportions. How this meta-inflammation affects HSCs and what long-term consequences it has on the innate immune system remains to be elucidated.

#### 3.1.2 Trained immunity a central IIM induced by acute HSC stimulation

##### 3.1.2.1 LPS and polyI:C

In contrast to the other previously mentioned stimuli, a single LPS stimulation leads to a transient activation of HSCs and HSPCs that returns to baseline within the first week (14). However, it leads to persistent epigenetic changes in the HSCs that last for at least 12 weeks. These epigenetically altered regions, called memory regions, specifically affect myeloid enhancers and genes of the innate immune system, making them more accessible. Upon re-exposure to LPS, the genes associated with the memory regions in the HSCs show a globally higher expression than after the first stimulation. Furthermore, transplantation of LPS- or polyI:C-pre-stimulated HSCs resulted in protective IIM against *Pseudomonas aeruginosa* through enhanced myeloid differentiation (14). Taken together, these results have shown that acute LPS or polyI:C stimulation are suitable models for studying central IIM. In contrast to IIM triggered by chronic stimuli, where the role of persistent epigenetic changes in the establishment of a protective memory against secondary infection is difficult to separate from the activation state of the cell, stimuli that trigger transient transcriptional reprogramming such as LPS allow a precise analysis of the role of epigenetic marks in the establishment of this memory.

##### 3.1.2.2 Heme

Heme, a component of hemoglobin that contains iron molecules and has short-lived immunomodulatory properties, has been shown to trigger an inflammatory response in macrophages via PRRs (61). Exposure to labile heme, as in sickle cell anemia in humans or after experimental injections in mice, protects against malaria (62) and polymicrobial sepsis (63), suggesting a possible role of IIM. Heme injections increase the production of proinflammatory cytokines, such as TNF-α, by BMDMs upon *ex vivo* LPS exposure and also enhance their phagocytic capabilities. These hallmarks of IIM depend on SYK/JNK-dependent acquisition of H3K27ac marks (63). Remarkably, no residual heme or cytokines were detected in the supernatant of macrophages prior to re-stimulation, indicating a return to steady-state and trained immunity. However, heme exposure in mice results in a sustained myeloid skew that begins at the earliest stage of hematopoiesis with an increase in myeloid-biased HSCs (CD41+) and MPP3. Heme also induced long-lasting changes in chromatin accessibility in HSCs, particularly in genes controlling myelopoiesis. Taken together, these results suggest that stimulation with heme leads to IIM establishment in mouse HSCs.

### 3.2 Duration and intensity: shaping function and memory?

Although many microorganisms, PAMPs and inflammatory cytokines induce myeloid differentiation of HSCs, the duration, intensity of HSC activation (exit of quiescence and proliferation) and functional outcome vary. The intensity of the HSC response may depend on the type of stimulus, its ability to trigger an inflammatory response in the bone marrow or to be recognized by the HSCs, and the number of receptors on the surface of the HSCs.

The threshold of inflammation that HSCs can sense appears to be very low. Indeed, HSCs show an even lower activation state at steady-state in absence of the IFN-γ receptor or when the microbiota has been compromised by antibiotics, suggesting that they can sense even low-grade inflammation (15, 64). It is not yet clear whether all responses including low-grade inflammation can trigger central IIM by leaving an epigenetic scar, and whether each modification enables an altered response to secondary infection or whether HSCs need to reach a certain level of activation to trigger IIM. Older studies on human monocytes have shown that the dose and thus the level of activation is important for the type of secondary response. Thus, high doses of LPS inhibit pro-inflammatory signaling pathways upon subsequent LPS stimulation, while low doses have the opposite effect (65). At the cellular level, this is likely similar, depending on how many receptors the cells express, how strong their response is, and which signaling pathways they can activate. In other words, HSCs are unlikely to mount the same IIM as progenitor cells or monocytes.

Furthermore, both acute and prolonged stimulation of HSCs can trigger IIM in HSCs, with the duration of stimulation playing a critical role in determining the outcome. While acute stimulation seems to have a reversible effect on HSC functions (10, 14, 31), chronic stimulation can lead to their depletion (11, 42, 66) and cause severe consequences such as pancytopenia. The type of stimulus also influences the impact on HSCs. For example, while transient activation by TNFα or IL-1β results in a temporary loss of function (10, 24), M-CSF does not affect their engraftment capacities (27), even after multiple injections (67, 68). In addition to the type of stimulus, bioavailability also modulates the effect on HSCs, as seen with a single exposure to β-glucan which induces a sustained activation of HSCs (13), unlike a single exposure to LPS or polyI:C (14, 69). Additionally, the number of stimuli is a critical factor. A recent study by Bogeska and colleagues demonstrated that repeated exposure of HSCs to chronic polyI:C treatment, interrupted by a 4-week recovery period, impairs HSC function even after an prolonged recovery period of 10 to 20 months. This impairment is characterized by reduced self-renewal capacity and functional potency of HSCs, accompanied by permanent myeloid bias and epigenetic alterations, as also observed in aged mice (69).

Therefore, it is important to consider the benefits of central IIMs in a context that may affect the long-term function of HSCs, such as the induction of chronic stimulation or accelerated aging.

### 3.3 Heterogeneity of HSC, heterogeneity of sensing? Heterogeneity of memory?

The era of single cell analysis has revealed significant heterogeneity within populations of phenotypically identical HSCs, both in steady state and in their responses to inflammatory signals. Under steady state conditions, this heterogeneity manifests itself in terms of differentiation into specific lineages, self-renewal and proliferation. These observations have been made using genetic models and single HSC transplantation (70, 71), as well as under homeostatic conditions using barcoding techniques (3, 4, 72). Intrinsic heterogeneity also extends to epigenetic (73) and metabolic (74) levels.

The question of whether all HSCs can respond to inflammatory stress and initiate IIM, most likely leading to heterogeneity, is of great interest to understand how inflammation can affect overall hematopoiesis by affecting the most primitive HSCs. It is still controversial whether primitive HSCs can respond to inflammatory signals. The use of proliferation tracking tools such as the H2B-GFP transgenic mouse model or the BrdU labeling retaining assay allows the distinction between LRCs (label-retaining cells or primitive HSCs) and non-LRCs (dividing/active HSCs). In response to LPS or polyI:C, even the most dormant HSCs that are LRCs, can be activated (21, 33). In contrast, the use of CD41, a marker reported to distinguish between myeloid/platelet-biased HSCs and balanced HSCs (75), reveals that HSCs responding to β-glucan or influenza are predominantly CD41+, while CD41- HSCs remain inactive (13, 39).

Regardless of their quiescent or activated state, single cells cultured *in vitro* and RNA-seq analyses have shown a heterogeneous response to various inflammatory stimuli such as mycobacteria, IL-1β, IFN-α and LPS. For example, *ex vivo* LPS or *in vivo* influenza exposure of single cells shows that the clonogenicity of HSCs varies with respect to output numbers and lineages (39, 76). Single-cell RNA-seq analyses following *ex vivo* exposure to LPS and PAM3CSK4 show a similar transcriptional response in HSCs and MPPs (77). Although all types of HSPCs can express ISGs following *in vivo* exposure to INFα or *M. avium*, they do so to varying proportions (20, 52). Overall, these studies suggest that HSCs respond differently depending on the type of exposure, likely due to the heterogeneity of receptor expression across all subtypes.

Although we have demonstrated that SLAM-HSCs can mount IIM in response to various stimuli (14), an analysis of *M. avium*-induced IIM in highly purified LT-HSCs expressing the EPCR marker–defined as the most primitive HSCs and sit at the top of the hematopoietic hierarchy – shows an inability to provide protection against secondary infection (52). This finding suggests that IIM cannot be initiated in EPCR+ LT-HSCs, unlike in SLAM-HSCs or that not all stimuli can trigger IIM in HSCs. The emergence of new technologies at the single cell level will allow a deeper understanding of IIM in HSCs, particularly in primitive HSCs.

### 3.4 Transfer of IIM from HSPC to downstream mature cells

Although the exact mechanism of how memory is transferred from cell to cell by divisions remains unknown, similar changes are observed between stimuli-exposed HSPCs and mature cells, suggesting that the underlying mechanisms of IIM are transferred from HSPCs to mature cells. Furthermore, functional assays show that exposure of HSPCs leads to mature cells with greater functions. Here we review the evidences for transmission of IIM from HSPCs to mature cells.

#### 3.4.1 Transfer to innate cells

##### 3.4.1.1 Transfer to monocytes/macrophages and neutrophils

Most studies in mice and humans addressing transmission of IIM from HSPC to mature cells have looked at myeloid cells, i.e. phagocytes. Monocytes/macrophages (mono/mac) and neutrophils in both humans and mice have a relatively short lifespan compared to other cells in the body. They circulate briefly in the bloodstream, typically only a few days for mono/mac and a few hours for neutrophils, before migrating into tissues where they fight infection. In tissues, their survival time ranges from a few days to a few weeks for mono/mac and from a few hours to a few days for neutrophils, depending on the presence of pathogens and the severity of inflammation (78). Despite this rather short half-life, mono/mac and neutrophils have been described as major effector cells of long-term IIM in response to e.g. BCG, β-glucan or LPS, suggesting that features of IIM might be transferred from HSPC to mature downstream effector cells.

In mice, functional transfer of IIM from HSCs to mature myeloid cells was demonstrated *in vivo* by transplanting whole bone marrow cells or purified HSPCs from BCG- (12), β-glucan- (43, 58) or LPS-exposed mice (14) into naïve recipients. Recipients were then allowed to stabilize for twelve to fourteen weeks to ensure that all mature cells were derived from the transplanted HSCs before new stimulation was initiated. Transplantation of T-cell-depleted bone marrow cells from BCG-exposed mice, protected the recipients against *Mycobacterium tuberculosis* (12). Similarly, transplantation of purified LPS-exposed HSPCs protected recipients against lethal *Pseudomonas aeruginosa* infection (14). In the case of β-glucan, transplantation of bone marrow from exposed mice resulted in protection against cancer development. Although a six-week delay between transplantation and secondary challenge may not be sufficient to demonstrate that the protective effect originates from LT-HSCs, it is at least sufficient to demonstrate the protective effect of MPPs and progenitor cells (58). Transfer of IIM has also been demonstrated *in vitro*. BMDMs were differentiated from the bone marrow of BCG-vaccinated mice and subsequently infected with *M. tuberculosis*. These macrophages derived from BCG-vaccinated bone marrow exhibited epigenetic changes, particularly the activation of enhancer elements, which increased the ability of macrophages from BCG-exposed bone marrow to mount a response to infection with *M. tuberculosis* (12).

Although the transfer of BCG-induced IIM from HSPCs to mature myeloid cells is also highly probable in humans, it has not been evaluated in a bone marrow or HSPC transplantation (48, 57). In healthy individuals vaccinated with BCG, PBMCs showed increased responsiveness to *Candida albicans in vitro*, even 90 days after vaccination, a period that extends beyond the typical lifespan of these cells. Furthermore, the transcriptomic changes induced by BCG vaccination in HSPCs are epigenetically transferred to larger short-lived PBMC-derived effector cells such as CD14+ monocytes, in part by regulating DNA accessibility at specific inflammation-associated loci. This mechanism enables the sustained induction of a memory phenotype in CD14+ monocytes (48) and neutrophils (57). At least in human CD14+ monocytes, the epigenetic modifications induced by BCG were similar to those found at the transcriptomic level in HSPCs, highlighting a crucial mechanism of IIM imprinting across cell lineages.

Interestingly, the transfer of IIM from HSPC only to neutrophils, but not to monocytes, has also been demonstrated in mice and zebrafish. This raises the intriguing and previously unresolved question of how innate memory can be either erased or selectively passed through differentiation from a conserved common progenitor only to neutrophils and not to monocytes.

In mice, this was demonstrated in a context where bone marrow from mice exposed to β-glucan for 7 days was transplanted into naïve recipient mice (58). The naïve recipients who received β-glucan-exposed bone marrow showed inhibition of melanoma tumor growth compared to those transplanted with naïve bone marrow. This was attributed to the presence of tumor-associated neutrophils (TAN), which produce higher levels of reactive oxygen species (ROS). In contrast, tumor-associated monocytes were not found to be involved in this process. Transcriptomic analysis of β-glucan exposed BM progenitor cells (GMP) and downstream mature cells in response to tumor revealed a common transcriptomic profile between GMP and neutrophils, but not monocytes. Although some of the transcriptomic changes were certainly due to the still ongoing activation of these cells induced directly by β-glucan, this indicated a transfer of transcriptomic changes via proliferation and differentiation from GMP to neutrophils. This indicates that in the context of anti-tumor immunity, neutrophils and granulocytic progenitors are the major cellular effectors of β-glucan-induced IIM.

IIM transfer from HSPCs only to neutrophils has also been demonstrated in zebrafish using advanced genetic tools and specific depletion systems targeting either monocytes or neutrophils. Furthermore, transplantation experiments have shown that primary infection with *Salmonella enterica* imprints IIM in HSPCs, which are subsequently transferred to downstream neutrophils but not to monocytes (79). The *Staphylococcus enterica*-exposed HSPC-derived neutrophils responded better – killing intracellular bacteria faster and producing more ROS – to a second infection with the same bacteria or with an unrelated pathogen such as *Streptococcus iniae* (79). These phenotypes are cell intrinsic as they are maintained after transplantation into infection-naive hosts. By studying the transcriptomes of exposed neutrophils, they identified an infection-induced transcriptional program that is likely responsible for increased mitochondrial ROS production and contributes to increased bactericidal activity. Another study by a different group also reported improved survival of *Shigella flexneri*-exposed larvae upon subsequent infection. This protection also correlated with an increase in the HSPC compartment, suggesting that this host response is not pathogen-specific, but rather a general protective mechanism (80), as can be concluded from studies in humans and mice.

##### 3.4.1.2 Transfer to dendritic cells

As for monocytes and neutrophils, despite their relatively short half-life, it is quite evident that dendritic cells can support the effector functions of IIM inherited from HSPCs, as they exhibit long-lasting effects that persist for extended periods of time, at least up to 70 days in mice (81). However, despite these observations, there is no formal demonstration of transplantation of pre-stimulated HSPCs into naïve recipients to conclusively demonstrate this link. This research gap leaves open the question of how effectively exposed HSPCs can reprogram dendritic cells in a naïve host and whether these exposed dendritic cells can independently sustain an enhanced immune response. Conducting such transplantation experiments would provide crucial insights into the mechanisms and persistence of IIM and the specific role that dendritic cells play in this process.

#### 3.4.2 Potential transfer to other blood cells?

IIM may also be transferred from HSC to other blood cells such as platelets and lymphocytes. For example, the transfer of IIM could occur in response to Influenza A virus (IAV) infection or to polyI:C, both stimuli inducing IIM in HSCs (14, 52). Transcriptomic analyzes of IAV- and polyI:C-activated HSCs revealed significant changes in gene expression, including enrichment in biological processes related to platelet production. Additionally, IAV-stimulated HSCs produce more platelets that are larger, have an immature phenotype and are hyper-reactive (39). Similarly, polyI:C stimulation causes HSCs to exit dormancy and enter the cell cycle, with some differentiating directly into megakaryocytes (21). Although the chromatin landscape of HSCs and megakaryocytes has not been studied, it is possible that IIM is also transferred from HSCs to megakaryocytes and then to platelets, which would be intriguing given the lack of chromatin DNA.

ILCs, NK cells and T cells also exhibit epigenetic memory after exposure, leading to enhanced secondary responses such as increased proliferation or reactivity, similar to IIM, as reviewed by Liotti et al. (82). Although HSCs usually do not show biased differentiation towards the lymphoid lineage, it is possible that they give rise to lymphoid cells harboring IIM after returning to a balanced state.

## 4 Beneficial versus detrimental impact of IIM

The first studies have shown several beneficial effects of IIM on mature cells. These include increased phagocytosis of monocytes and macrophages in response to secondary infections (12, 48, 52, 55–57, 65), a reduction in dust-induced asthma (83, 84), antitumor effects (58, 85) or protection against chemotherapy-induced DNA damage (13). However, there is increasing evidence that IIM can also have unfavorable consequences, often referred to as maladaptive trained immunity. The maladaptive response can arise from deleterious memory effects, as mentioned above with *Mycobacterium tuberculosis* (43), or from a memory that is beneficial in response to one secondary stimulus and detrimental to another. In addition, an out-of-control memory response can also lead to adverse outcomes.

It remains unclear how memory retrieval works in response to different secondary exposures. On the one hand, as described in a previous section, IIM induced by *Mycobacterium avium* can be reactivated by secondary infection with *Mycobacterium avium* or by influenza, leading to reduced pathogenicity in both scenarios (52). However, the inflammatory cytokine profiles are opposite in these two cases, suggesting that different mechanisms underlie the IIM recall. On the other hand, IIMs can be either beneficial or detrimental to the organism depending on the secondary stimulation. For example, both β-glucan/BCG (13, 86) and oxLDL/western diet (17) have a similar IIM mechanism that depends on IL-1β and a metabolic switch that allow an increase in TNF-α and IL-6 in response to LPS. However, in the case of β-glucan/BCG, this mechanism provides protection against subsequent infection with *Mycobacterium tuberculosis*, whereas in the case of oxLDL/Western diet, it promotes diabetes and atherosclerosis.

IIM is a tightly controlled process that allow an enhanced/dampened response only upon a second exposure. For instance, the first exposure to BCG or β-glucan allows an increase in the pro-inflammatory cytokines TNF-α and IL-6 only when the cells are stimulated again with LPS. However, in some cases, pro-inflammatory cytokines are continuously produced even without secondary stimulation, suggesting that IIM is out of control. This has been shown to promote or exacerbate pro-inflammatory diseases such as atherosclerosis, SLE (systemic lupus erythematosus), sarcoidosis or arthritis as reviewed in (87, 88). Furthermore, these inflammatory diseases also occur in patients with a clonal hematopoiesis, particularly CHIP (clonal hematopoiesis of indeterminate potential). Clonal hematopoiesis is a process in which certain HSC clones become dominant, which is often observed during aging. Although not yet proven, it is hypothesized that HSCs from CHIP could harbor an out-of-control IIM may be the source of proinflammatory cells that cause chronic inflammation (89).

## 5 Intrinsic drivers, and underlying molecular mechanisms of immune memory

**Figure 4:**
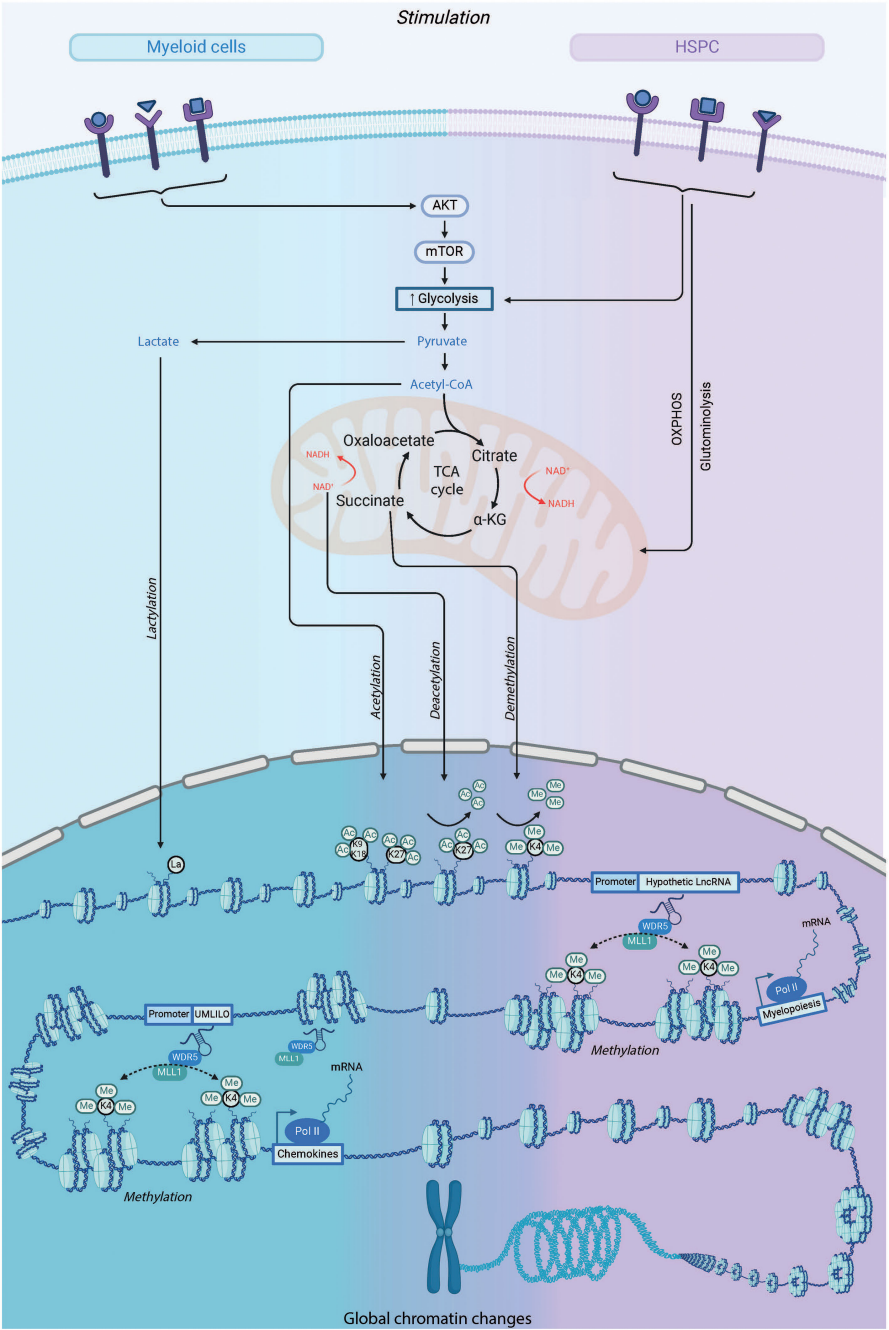
Metabolic and Epigenetic Pathways involved in IIM: A Comparative Insight into HSPCs vs. Myeloid Cells. The initiation of mechanisms required for inducing innate immune memory depends on the duality of epigenetic and metabolic reprogramming of innate immune cells upon stimulation. During the initial challenge, the recognition of specific ligands by patter recognition receptors triggers a series of intracellular cascades, leading to the upregulation of various metabolic pathways such as glycolysis, the tricarboxylic acid (TCA) cycle, and glutaminolysis. Metabolites produced from these processes, like fumarate and acetyl coenzyme A (acetyl-CoA), can activate or inhibit enzymes involved in remodeling the cell's epigenetic landscape, such as the histone demethylase lysine-specific demethylase 5 (KDM5) and histone acetyltransferases. This results in specific changes in the histone methylation and acetylation of genes involved in innate immune responses. This increases the accessibility of DNA to the transcriptional machinery and gene regulatory elements, as well as specific long non-coding RNAs, thereby promoting and facilitating enhanced gene transcription upon secondary stimulation of the cells.

The exact molecular mechanisms responsible for the altered responsiveness of IIM-bearing cells are not fully understood. However, the available evidence suggests that several regulatory pathways converge to influence this phenomenon. These pathways include reprogramming of cellular metabolism, changes in chromatin organization within topologically associated domains (TADs) and transcription of long non-coding RNAs (lncRNAs). This reprogramming facilitates conserved epigenetic histone modifications that act like DNA scars that can be reopened upon *de novo* stimulation (**Figure 4**). Another layer of intrinsic factor essential for IIM is the role of transcription factors (TFs). These mechanisms have been extensively studied in mature myeloid cells (as reviewed (90)), but are still poorly understood at the HSC level.

### 5.1 Immunometabolic reprogramming

Historically, IIM-associated metabolic reprogramming have been massively investigated in mature myeloid cells. To date, however, some mechanisms also appear to be shared in HSPCs. The metabolic state of the cells is a crucial clue to determine their activation status. HSCs primarily remain in a dormant state to avoid premature exhaustion due to replicative stress (91). The hypoxic niche in which they reside forces HSCs to rely on anaerobic glycolysis for their survival (92, 93). They also have low metabolic activity, which is associated with low protein production and reduced mitochondrial activity (94). After activation and exit from the quiescent state, they undergo major metabolic changes. In particular, they show increased metabolic activity for both glycolysis and OXPHOS (95–97), a conserved feature for many cells. Indeed, inhibition of mitochondrial respiration in HSCs has been shown to lead to a rapid defect in hematopoiesis in bones (98). The mTOR-AKT signaling pathway is a key factor in glucose uptake and appears to be crucial in BCG and β-glucan-induced IIM (99, 100), as blockade of this pathway inhibits proinflammatory secretion induced by LPS in monocytes (99, 100). In HSPCs, treatment with β-glucan resulted in enhanced myelopoiesis, which was accompanied by increased metabolic activity, particularly glycolysis, suggesting that the same mechanism occurs in mature myeloid cells and HSPCs (13). Consequently, increased glucose uptake is accompanied by an increase in pyruvate and lactate production. Increased intracellular lactate is associated with lactylation of lysine residues on histones (101), whereas pyruvate drives both mitochondrial respiration and the pentose phosphate pathway. Albeit the pentose phosphate pathway does not appear to be involved in IIM (13, 99), mitochondrial respiration is known to provide some key elements. After the conversion of pyruvate to acetyl-CoA, the molecule is now able to enter the tricarboxylic acid (TCA) cycle. In addition, acetyl-CoA is a key factor for histone acetyl transferases (HATs) and thus enables epigenetic acetylation. Fumarate and succinate, 2 derivatives of α-ketoglutarate, also act as competitive inhibitors of histone demethylases such as histone demethylase KMD5. After treatment with β-glucan, human monocytes showed an increase in fumarate and succinate, which was accompanied by increased glycolysis (100). This increase in metabolism is associated with decreased KMD5 histone demethylase and therefore increased H3K4me3 histone mark (99).

Another consequence of increased glycolysis is a high ratio of nicotinamide adenine dinucleotide (NAD+) to its reduced form (NADH). NAD+ is a crucial cofactor for the activity of all seven mammalian sirtuins (SIRTs), a group of enzymes that remove acetyl groups from lysine on histones (102). Consequently, NAD+ concentration enables the SIRTs to regulate the degree of histone acetylation, which is a key factor in the control of IIM. In HSCs, sirtuins have been demonstrated to play a central role in genomic stability related to stress, quiescence and regenerative capacity (103). It has been shown that deletion of SIRT6 in adult mouse HSCs results in an increase in proliferation, accompanied by an increase in H3K56ac, but with a significant impairment in their long-term repopulation capacity (104). It is therefore possible that both NAD and SIRT proteins play a key role in central IIM.

### 5.2 Epigenetic changes upon trained immunity

Metabolism and epigenetics form the dual foundation of IIM and are in a constant exchange of influences. Within this intricate relationship, various previously described metabolic pathways act as a constant source of energy and essential components that drive the dynamic reconfiguration of the cellular epigenetic landscape. At the same time, they provide the necessary materials to shape the structure of chromatin and the genome. In the context of this remodeling, we observe an altered functional responsiveness associated with altered chromatin accessibility, which is one of the hallmarks of IIM.

Histone methylation plays a central role in the HSPC compartment in response to various stimuli. They are often localized at specific lysines of histone 3 such as H3K4, H3K27, H3K9 or more recently H3K36 and are globally associated with gene repression or permissive chromatin structure. Numerous enzymes and complexes influence the degree of lysine methylation, such as G9a or EZH2. Histone lysine residues can be mono- (m1), di- (m2) or trimethylated (m3), and these modifications are distributed differently across the chromatin, each playing distinct roles in gene regulation. For example, H3K4me1 and H3K4me2 or 3 mark enhancer and promoter positions, respectively. In addition, it is known that acetylation of H3K27 changes the chromatin structure by neutralizing the histone charge, which leads to an opening of the chromatin.

Key epigenetic marks involved in IIM in myeloid cells include increased accessibility of enhancers and promoters through the acquisition of H3K27ac marks at distal enhancers and the maintenance of H3K4me3 marks at the promoters of memory genes (105). In contrast, the promoters of tolerized genes remain deacetylated and inaccessible (106).

There is compelling evidence that BCG stimulation induces epigenetic, transcriptional and functional changes in BMDMs for up to five months. Indeed, H3K27Ac levels were altered at thousands of sites that mainly overlap with enhancers, and these changes were correlated with genes that have effector functions in BMDMs (12). In a separate study in monocytes, BCG was observed to increase H3K27Ac levels in several regions associated with increased production of growth factors (VEGF, EGFR and FGF) and cytokines (86). Finally, our laboratory demonstrated that chromatin accessibility near H3K4me1 marks specific for myeloid cells in LT-HSC was increased after LPS stimulation, although gene expression was not altered. This suggests that myeloid enhancers are poised in LT-HSCs to favor their differentiation and protection against subsequent *Pseudomonas aeruginosa* infection (14).

Mouse macrophages stimulated with LPS have been shown to have a high concentration of lactate capable of binding histones via a mechanism called “histone lactylation” (101). The generation of lactylated histones is associated with increased expression of genes responsible for cellular homeostasis and decreased expression of proinflammatory genes (101). Recently, in a glioblastoma mouse model, monocyte-derived macrophages (MDM), which are known to be the major immunosuppressive cells of this tumor microenvironment, were found to have high levels of lactylated histone, which is associated with an immunosuppressive phenotype (107). Disrupting glucose uptake of MDMs by targeting PERK abrogates histone lactylation (107). Thus, histone lactylation appears to be a regulatory mechanism that balances the expression of inflammatory genes at the epigenomic level. The acetyltransferase enzyme p300, which is known to be important for H3K27Ac, is proposed as a potential scribe protein for histone lactylation (101). In this study, specific inhibition of p300 abrogates this mechanism (107), making this enzyme critical for IIM.

Overall, these results provide strong evidence supporting the crucial role of an epigenetic modifier in the mechanism of trained immunity in mature cells and HSC.

### 5.3 The role of long non-coding RNA in trained immunity: investigating 3D nuclear structure involvement

Another mechanism by which cells may be epigenetically imprinted is the action of lncRNAs. They are defined as transcripts that are longer than 200 nucleotides and are not translated into proteins. It is well documented that an increasing number of long non-coding RNAs (lncRNAs) play an important role in cells, notably by affixing epigenetic marks involved in the formation of IIM. The expression of many lncRNAs is subject to regulation, and some are involved in various mechanisms of gene regulation. Numerous lncRNAs regulate the expression of nearby genes (cis) and influence their transcription, but others are able to act distally (trans) from their genomic location (108). To this end, they are organized in a 3D loop structure (109) called topological association domains (TADs).

Extensive work to understand lncRNA in the hematopoietic compartment has shown that they play a role in the progression of hematopoiesis (110). In mature immune cells, one of these lncRNAs, called UMLILO, has been shown to facilitates laying of H3K4me3 epigenetic mark by the methyltransferase complex WDR5-MLL1 at the *Cxcl* genes promoters of mouse macrophages in response to TNFα and β-glucan (111). This leads to increased expression of the associated *Cxcl* genes upon subsequent stimulation with LPS. Importantly, inhibition of UMLILO completely resets the epigenetic modifications conferred by β-glucan, suggesting a key role in IIM.

On the other hand, lncRNAs in HSCs have been identified as important for the differentiation process and lineage decision of HSCs, making them interesting for analyzing the establishment of IIM in HSCs. It has been described that lncHSC-1 is exclusively present in HSCs, whereas lncHSC-2 is present in both HSCs and progenitor cells. Both have opposite functions. LncHSC-1 enhances myeloid differentiation whereas lncHSC-2 promotes lymphoid production (112, 113). To some extent, lncHSC-2 also appears to play a role in the self-renewal capacity of HSCs. To date, it has not been shown that an lncRNA epigenetically marks HSCs in the same manner as UMLILO marks mature myeloid cells. However, it is strongly suspected that this mechanism operates in bone marrow cells after BCG stimulation, as they show epigenetic rewiring, in particular an enrichment of H3K4me3 in genes involved in IIM (12).

Understanding the 3D core structure is crucial for a comprehensive understanding of the intricacies of HSC differentiation and how lncRNAs influence the central imprinting of immune memory observed in mature myeloid cells.

### 5.4 Transcription factors involved in central IIM

TFs are required for various cellular processes such as differentiation and response to environmental signals. They function by recognizing a specific DNA motif in promoter or enhancer regions and activating or inhibiting the transcription of the associated genes by recruiting the transcription machinery or proteins for chromatin remodeling. Pioneer factors are specific TFs that have the ability to bind to closed chromatin and promote its remodeling to open it up and make it accessible to other factors and gene expression. This makes them important players in cellular differentiation and reprogramming. In this sense, they appear to be very important for the establishment of IIM. Other TFs may also be involved in the enhanced/dampened function of cells without being involved in epigenetic reprogramming.

The pioneer and myeloid TF C/EBPβ (114) is enriched in memory regions induced by LPS in HSCs. The memory regions were completely absent in HSCs lacking C/EBPβ, suggesting that C/EBPβ is a key factor in the establishment of LPS memory in these cells (14). This was confirmed by Larsen et al. with a further level of analysis, as they also highlighted the importance of TFs Fos and Jun, members of the AP1 family (115). Thus, both studies suggest that C/EBPβ as a pioneer factor may enable chromatin opening in response to LPS and facilitate access to Jun and Fos that may be required for the maintenance of memory domains. In addition, motifs of CEBP family members were also enriched in open chromatin regions of HSPC from the blood of patients severely affected by COVID-19 months after disease onset or in HSCs from mice after MHV-1 infection (26). In the latter study, Fos and Jun were enriched only in the first months after disease onset and not in late convalescence, suggesting an alternative mechanism in the long-term maintenance of memory domains (26). Finally, in zebrafish, C/EBPβ signaling induced during infection with *Staphylococcus enterica* or overexpression of ectopic C/EBPβ in naïve HSPCs is sufficient to drive this hematopoietic program and produce neutrophils with similarly enhanced bactericidal activity (79) all suggesting that C/EBPβ is an essential mediator of IIM in HSPCs.

Other TF families may also be important for the epigenetic memory of HSCs, such as those enriched in the memory regions of LPS-trained HSCs, like PU.1, STAT, ATF or KLF (14), and some of which also play an important role in epigenetic remodeling and training of myeloid cells (116, 117). In monocytes and macrophages, the most studied TF involved in IIM is the master regulator of the hypoxia response HIF1. Inhibition or depletion of HIF1 in myeloid cells completely abrogates β-glucan-induced rewiring by disrupting the metabolic switch required for epigenetic reprogramming (99, 100). HIF1 is also very important for the balance between the quiescent and proliferative state of HSCs (92). It would therefore be very interesting to know whether it also plays a role in the epigenetic reprogramming of HSCs after inflammation.

## 6 Conclusion and future perspectives

In recent decades, the discovery of the immune innate memory has opened new avenues for therapeutic approaches that provide comprehensive protection against many infections, but also against other diseases such as cancer or in transplantation in immunocompromised patients.

We have emphasized here that HSCs, as important sensors of inflammation, promote emergency myelopoiesis at the very beginning of hematopoiesis. Moreover, the induced response leaves permanent epigenetic marks in these cells that trigger IIM. Associated genes are activated or silenced, and the HSCs have enhanced or diminished functions upon a second encounter, resulting in a beneficial or detrimental response. Although the mechanism of how IIM is passed on to progeny despite epigenetic remodeling during differentiation is still unclear, mature cells from the bone marrow of exposed mice show epigenetic rewiring and enhanced clearance of pathogens.

In this review, we have focused on infectious stimuli such as microorganisms, PAMPs and inflammatory cytokines that can activate the response of HSCs and induce IIM in these cells, but there are other causes of an inflammatory environment such as cancer or certain lifestyles that could also be recognized and reprogram HSCs. Some of these stimuli may induce persistent activation of HSCs and we would like to draw attention to the fact that they can alter the regenerative capacity of bone marrow HSCs and possibly their exhaustion. In addition, the induction of sustained myeloid differentiation triggered by some stimuli is a hallmark of aging and may affect long-term lymphopoiesis. Therefore, it is very important to consider all properties of HSCs when analyzing stimuli for potential IIM therapeutic approaches, as well as the potentially deleterious effects induced by maladaptive trained immunity. For this reason, we have proposed to distinguish between IIM induced by acute and chronic stimulation.

In summary, HSCs, as long-lived cells that can pass IIM to myeloid cells during differentiation, are a reservoir for IIM. As they are at the top of the hematopoietic hierarchy, further research is needed to fully understand whether IIM can affect the entire hematopoietic system or whether some unresponsive HSCs are able to maintain naïve hematopoiesis over time.
